# CD8α+ dendritic cells dictate immune responses against murine AML

**DOI:** 10.1186/2051-1426-1-S1-P158

**Published:** 2013-11-07

**Authors:** Douglas E Kline, Dominick Fosco, Xiufen Chen, Justin Kline

**Affiliations:** 1 Committee on Immunology, University of Chicago, Chicago, IL, USA; 2 Department of Medicine, University of Chicago, Chicago, IL, USA

## 

Spontaneous T cell responses generated against a variety of solid malignancies are often subverted by immune evasion mechanisms active in the tumor microenvironment. In contrast, the mechanisms that regulate T cell activation versus tolerance to hematopoietic malignancies, such as acute myeloid leukemia (AML), have not been well-characterized. Our recent work in a murine AML model has demonstrated that following a systemic introduction of leukemia cells, T cells specific for leukemia-derived antigens underwent abortive proliferation and were deleted from the host. This deletional tolerance in mice with established AML was reversible upon administration of an agonistic anti-CD40 antibody to activate host dendritic cells (DCs), and argued that these cells may play a dominant role in tolerance induction to AML. Investigation of the DCs populations which engulfed AML cells in vivo, and which were likely promoting T cell tolerance, led to the critical observation that AML cells were phagocytosed exclusively by CD11c+CD8α+ DCs (CD8α+ DCs). CD8α+, but not CD8α- DCs purified from mice following an intravenous inoculation of AML cells, were able to cross-present leukemia-derived antigens to T cells in vitro, providing strong evidence that CD8α+ DC generate T cell tolerance to AML. Ongoing work utilizing mice deficient in particular DC subsets is focused on identifying a functional link between CD8α+ DCs and T cell tolerance. Additionally, the receptors expressed selectively on CD8α+ DCs which facilitate phagocytosis and cross-presentation of leukemia derived antigens are under investigation.

